# Karyotype evolution in *Phalaris* (Poaceae): The role of reductional dysploidy, polyploidy and chromosome alteration in a wide-spread and diverse genus

**DOI:** 10.1371/journal.pone.0192869

**Published:** 2018-02-20

**Authors:** Grit Winterfeld, Hannes Becher, Stephanie Voshell, Khidir Hilu, Martin Röser

**Affiliations:** 1 Institute of Biology, Martin Luther University Halle-Wittenberg, Halle (Saale), Germany; 2 Institute of Evolutionary Biology, University of Edinburgh, Edinburgh, United Kingdom; 3 Department of Biological Sciences, Virginia Tech, Blacksburg, Virginia, United States of America; Università di Pisa, ITALY

## Abstract

Karyotype characteristics can provide valuable information on genome evolution and speciation, in particular in taxa with varying basic chromosome numbers and ploidy levels. Due to its worldwide distribution, remarkable variability in morphological traits and the fact that ploidy change plays a key role in its evolution, the canary grass genus *Phalaris* (Poaceae) is an excellent study system to investigate the role of chromosomal changes in species diversification and expansion. *Phalaris* comprises diploid species with two basic chromosome numbers of *x* = 6 and 7 as well as polyploids based on *x* = 7. To identify distinct karyotype structures and to trace chromosome evolution within the genus, we apply fluorescence *in situ* hybridisation (FISH) of 5S and 45S rDNA probes in four diploid and four tetraploid *Phalaris* species of both basic numbers. The data agree with a dysploid reduction from *x* = 7 to *x* = 6 as the result of reciprocal translocations between three chromosomes of an ancestor with a diploid chromosome complement of 2*n* = 14. We recognize three different genomes in the genus: (1) the exclusively Mediterranean genome A based on *x* = 6, (2) the cosmopolitan genome B based on *x* = 7 and (3) a genome C based on *x* = 7 and with a distribution in the Mediterranean and the Middle East. Both auto- and allopolyploidy of genomes B and C are suggested for the formation of tetraploids. The chromosomal divergence observed in *Phalaris* can be explained by the occurrence of dysploidy, the emergence of three different genomes, and the chromosome rearrangements accompanied by karyotype change and polyploidization. Mapping the recognized karyotypes on the existing phylogenetic tree suggests that genomes A and C are restricted to sections *Phalaris* and *Bulbophalaris*, respectively, while genome B occurs across all taxa with *x* = 7.

## Introduction

It has become increasingly apparent that changes in chromosome number by polyploidy or dysploidy and structural rearrangements like inversions, deletions, or translocations play a substantial role in the evolution of many plant lineages [1, 2, 3, 4]. Further mechanisms like homoploid hybrization or polyploidization were considered as essential contributors to altered chromosomal behaviours [5, 6]. However, for a detailed historic assessment of chromosomal patterns within a group and their correlation with morphological innovations and geographic expansion, a valid phylogenetic hypothesis is essential [7]. The genus *Phalaris* L. (Poaceae) stands out as an excellent system to study the types and extent of chromosomal changes and their role in species diversification and expansion due to its pattern of global species distribution, presence of different basic chromosome numbers and ploidy levels, varied morphological characters, and the availability of robust phylogenetic and phylogeographic hypotheses [8, 9, 10].

*Phalaris* comprises 20 annual and perennial species that are distributed in temperate areas of both hemispheres and in the mountains of tropical Africa and South America [8, 9, 11]. *Phalaris* is taxonomically placed within the Aveneae-Poeae tribe complex or the expanded tribe Poeae of the subfamily Pooideae [12, 13, 14, 15, 16, 17]. Infrageneric treatment based on molecular phylogenetics, floret structure, cytology and biogeographic distribution [9] established two subgenera (*Phalaris* and *Phalaroides*) and five sections (*Phalaris*, *Phalaroides*, *Caroliniana*, *Bulbophalaris*, *Heterachne*). Of its wild species, 18 have been studied chromosomally, twelve are exclusively diploid but reveal two different chromosome basic numbers of *x* = 6 and *x* = 7 (2*n* = 12 and 14 [18, 19, 20]). Six are polyploid, five of them are tetraploid (2*n* = 4*x* = 28), and one (*P*. *caesia* L.) is hexaploid (2*n* = 6*x* = 42; summarized in [10]). The hybrid *P*. *arundinacea* × *P*. *aquatica* is octoploid with 2*n* = 8*x* = 56 [21]. Karyotype studies in *Phalaris* further than mere chromosome counting concerned traditional karyotyping and C-banding techniques [22], physical mapping of 45S and 5S rDNA sites by fluorescence *in situ* hybridization [23] or studies of the meiotic behaviour [24]. In molecular phylogenetic analyses, Voshell [8] included 20 species of *Phalaris* and used nuclear ribosomal ITS and plastid *trnT-F* sequences. This study established a single origin of the genus, revealed a lineage of three taxa with *x* = 6 which all belong to subgenus *Phalaris* section *Phalaris* and established the sister relationship of this lineage to the three monophyletic and more species-rich lineages with *x* = 7 [18, 19, 20] of section *Phalaroides*, *Caroliniana*, and *Bulbophalaris* + *Heterachne* (subgenus *Phalaroides*; [8, 9]. Associations of changes in basic chromosome number with morphological modifications especially in the number and dimension of the sterile lemmas were shown within the genus [21, 8]. Members of the early diverging *x* = 6 lineage displaying relatively large and lanceolate sterile lemmas, followed by gradual reduction in size, culminating in almost obsolete sterile lemmas in one of the terminal *x* = 7 clades. In addition, the clades recovered in the molecular analyses of Voshell [8] display a geographic structure. They concur with the results of Baldini [25] who recognized two main centres of differentiation. One of them occurs in the Old World Mediterranean area, comprising the *x* = 6 taxa of subgenus *Phalaris* and the *x* = 7 taxa of subgenus *Phalaroides* sections *Phalaroides*, *Heterachne* and *Bulbophalaris*. The other occurs in the New World southwestern USA region comprising the *x* = 7 taxa of sections *Caroliniana* and *Phalaroides*. Some secondary centres of differentiation exist in Africa, Asia and the Americas [10]. According to these studies, the genus should have been established during the Miocene (20.6–8.4 million years ago) in the Mediterranean Basin. It has been hypothesized that the evolution within the genus may have initiated from a supposed and unknown diploid ancestor with a 2*n* = 14 like *P*. *rotgesii* [25] or *P*. *arundinacea* [10]. Subsequent dispersal and vicariance events to secondary centres in Africa, Asia and the Americas were accompanied by chromosome fusion leading to species with *x* = 6, by genetic mutations and chromosomal changes in *x* = 7 diploids, and the establishment of polyploid species with 2*n* = 4*x* = 28 and 6*x* = 42 chromosomes [19, 20, 21, 25, 26, 27]. While polyploidy played a major role in the evolution of the genus in the Old World, the New World species remained primarily diploid.

Although information on *Phalaris* chromosome numbers and ploidy levels as well as on chromosome aberrations, aneuploidy and dysploidy was gathered occasionally [10, 18, 19, 20, 21, 24, 25], the species of *Phalaris* have never been characterized karyotypically in detail. Additionally, due to the small number of species studied and the inconsistency of the methods used in studies, only marginal evidence on karyotype evolution within the genus was available so far. Up to now, none of the hypotheses on the chromosomal evolution of *Phalaris* species could provide consistent information about the structure of an ancestral karyotype, the mode of basic number reduction, karyotype differentiation and the mode of genome composition in the polyploids. Fluorescence *in situ* hybridisation technique has been widely applied for the analysis of parental genomes in hybrids and auto/allopolyploids, and also for chromosome evolution in closely related species [28, 29, 30]. Specific repetitive sequences such as the tandemly repeated 5S and 45S rDNA have been used in numerous studies as cytogenetic marker [31, 32, 33, 34] to compare the cytogenetic positions of 5S and 45S rDNA sites among relative species. Consequently, in this study the karyotypes of the different *Phalaris* species were investigated and the distribution of the 45S rDNA and *5*S rDNA sites were mapped physically by FISH on the chromosomes of eight selected species covering all clades of the phylogenetic tree built by Voshell [8].

The aim of this study was to characterize the karyotype of the *Phalaris* species in order to (1) trace the pathway of basic number reduction and its impact on genome size, (2) investigate the potential occurrence of different genomes and kinds of polyploidy (alloploidy, autoploidy) that played a role in the evolution of the genus, and 3) characterize a hypothetical ancestral karyotype and the subsequent mechanisms of chromosome rearrangements within an available phylogenetic framework. These data should provide information about the importance of chromosomal reshuffling for species diversification, reproductive isolation and speciation. We will discuss the results in the framework of species distribution patterns and possible expansion routes within and between the New and the Old Worlds.

## Material and methods

### Plant material

Seeds of *Phalaris* species used in this study were provided by the USDA National Plant Germplasm System (*P*. *aquatica*, *P*. *canariensis*), or obtained from seed exchange with the Botanical Garden Tver, Russia (*P*. *arundinacea*), the Millennium Seed Bank, Wakehurst Place, UK (*P*. *brachystachys*, *P*. *caroliniana*), the Botanical Garden Berlin-Dahlem, Germany (*P*. *coerulescens*), and the Botanical Garden Frankfurt a.M., Germany (*P*. *minor*, *P*. *paradoxa*). The provenances, collectors and handling numbers are listed in Table 1. Caryopses of *Phalaris* species were germinated on Petri dishes with 1% Agar in a controlled environment under long-day conditions at 20°C. The plants were later grown in pots with prepared soil (compost:sand 2:1) in greenhouse at 20°C.

**Table 1:** Accessions studied with collection details and main quantitative results on chromosome characters evaluated. Vouchers are deposited at the herbaria HAL, handling number A = J. Schneider, GW = G. Winterfeld, R = M. Röser, SBD = Seed bank database. Karyological parameters: 2*n* = Ploidy level (*x*) and chromosome number of somatic cells; TML = Total Monoploid Length of chromosome set; M_CA_ = Mean Centromeric Asymmetry, CV_CL_ = Interchromosomal Asymmetry, 45S = Number of 45S rDNA signals / chromosomes with signals, 5S = Number of 5S rDNA signals / chromosomes with signals.

Subgenus/Section	Taxon	Provenance, collector and handling number	Genome	2*n* =	TML [μm]	M_CA_	CV_CL_	45S	5S
*Phalaris/Phalaris*	*P*. *brachystachys* Link	Greece, Peloponnisos; SBD 032915; GW151	A	2*x* = 12	50.2	25.5	18.4	2/2	4/2
	*P*. *canariensis* L.	Morocco; Hilu &Voshell; R902 (HAL140596)	A	2*x* = 12	35.0	31.4	13.3	2/2	4/2
*Phalaroides/Bulbophalaris*	*P*. *aquatica* L.	Greece; Hilu & Voshell (as *P*. *minor*), R900 (HAL140601)	B/C	4*x* = 28	49.6	9.9	11.8	4/4	12/7
			B		49.3	10.5	15.0	2/2	2/2
			C		49,9	9.4	9.9	2/2	10/5
	*P*. *minor* Retz	-, Seeds from Botanical Garden Frankfurt a.M.; R910 (HAL140600)	B/C	4*x* = 28	38,9	8,6	25.8	2/2	16/14
	*P*. *paradoxa* L.	-, Seeds from Botanical Garden Frankfurt a.M.; R908 (HAL140599)	B	2*x* = 14	30.3	9.2	14.7	2/2	2/2
*Phalaroides/Caroliniana*	*P*. *caroliniana* Walt.	USA, Texas; SBD 0386777; GW154	BB	4*x* = 28	47.6	8.2	13.1	4/4	4/4
*Phalaroides/Heterachne*	*P*. *coerulescens* Desf.	Italy; seeds from Botanical Garden Berlin-Dahlem; R907 (HAL140318, HAL 140319)	B	2*x* = 14	49.6	21.3	12.6	2/2	3/3
			B		28.8	18.5	18.9	2/2	4/4
			C		40.0	19.3	12.4	---	12/10
*Phalaroides/Phalaroides*	*P*. *arundinacea* L.	Russia, Staritza region, seeds from Botanical Garden Tver; A12	BB	4*x* = 28	46.0	12.1	15.4	4/4	2/2

### Chromosome preparation

For chromosome preparation and the subsequent fluorescence *in situ* hybridization (FISH), young growing root tips were immersed in distilled water, cold treated at 0°C for 24 h to accumulate metaphases, fixed in freshly prepared 1:3 glacial acetic acid:absolute ethanol for at least 3 h and stored in absolute ethanol at -20°C until preparation. Root tips were softened in an enzyme solution of 1% cellulase (w/v) and 10% pectinase (v/v) in citric acid-sodium citrate buffer pH 4.8 for 2 h at 37°C [35]. Squash preparations were made on slides in a drop of 45% propionic acid with 2% carmine. Preparations were analyzed under a Zeiss Axioskop 2 microscope and chromosome images with well-spread metaphases were captured using a computer-assisted cooled CCD camera (Zeiss Axiocam HRC) employing Zeiss Axiovision software.

### DNA probes

For FISH, total genomic DNA was isolated from silica gel-dried fresh leaves of *Melica ciliata* (HB15/1) using the NucleoSpin Plant II Kit of Marcherey-Nagel (Düren, Germany) according to the manufacturer’s instructions. The amplification of specific DNA regions was carried out with the 5S rDNA primers 5SGW (GAG AGA GTA GTA CTA GGA TGG) and p5S1 (CGC TTA ACT TCG GAG TTC TGA TGG) and the primers for regions of the 45S rDNA 18S-F (CCA GTA GTC ATA TGA TTG TCT C) and 18S-R (AGG TTC ACC TAC GGA AAC C). Amplifications were performed on an Eppendorf Mastercycler (Hamburg, Germany) using a reaction mix containing 0.5 *μ*M each of forward and reverse primer, 2 *μ*l of 10×Taq specific buffer (Quiagen, Benelux B.V., The Netherlands), 1.75 mM MgCl_2_, 1 U Taq DNA polymerase (Qiagen), 200 *μ*M dNTPs (GeneCraft, Lüdinghausen, Germany), 1 ng/μl of template DNA and an aliquot of purified water to obtain a final volume of 20 *μ*l. The PCR program was for 5S: 55min [94°C 3 min, (94°C 30 s, 55°C 30 s, 72°C 5 min) × 35, 72°C 1 min, 8°C stop] and for 18S: [94°C 3 min, (94°C 30 s, 51°C 30 s, 72°C 2 min) × 35, 72°C 1 min, 8°C stop]. PCR products were column-purified with the NucleoSpin Gel and PCR clean-up Kit of Macherey-Nagel and dissolved in H_2_O. The probes were labelled with fluorescein-dUTP (45S rDNA)- and tetramethylrhodamine-dUTP (5S rDNA) using the Nick-Translation Mix of Roche Diagnostics (Mannheim, Germany) according to the manufacturer’s instructions.

### FISH

**Fig 1:**
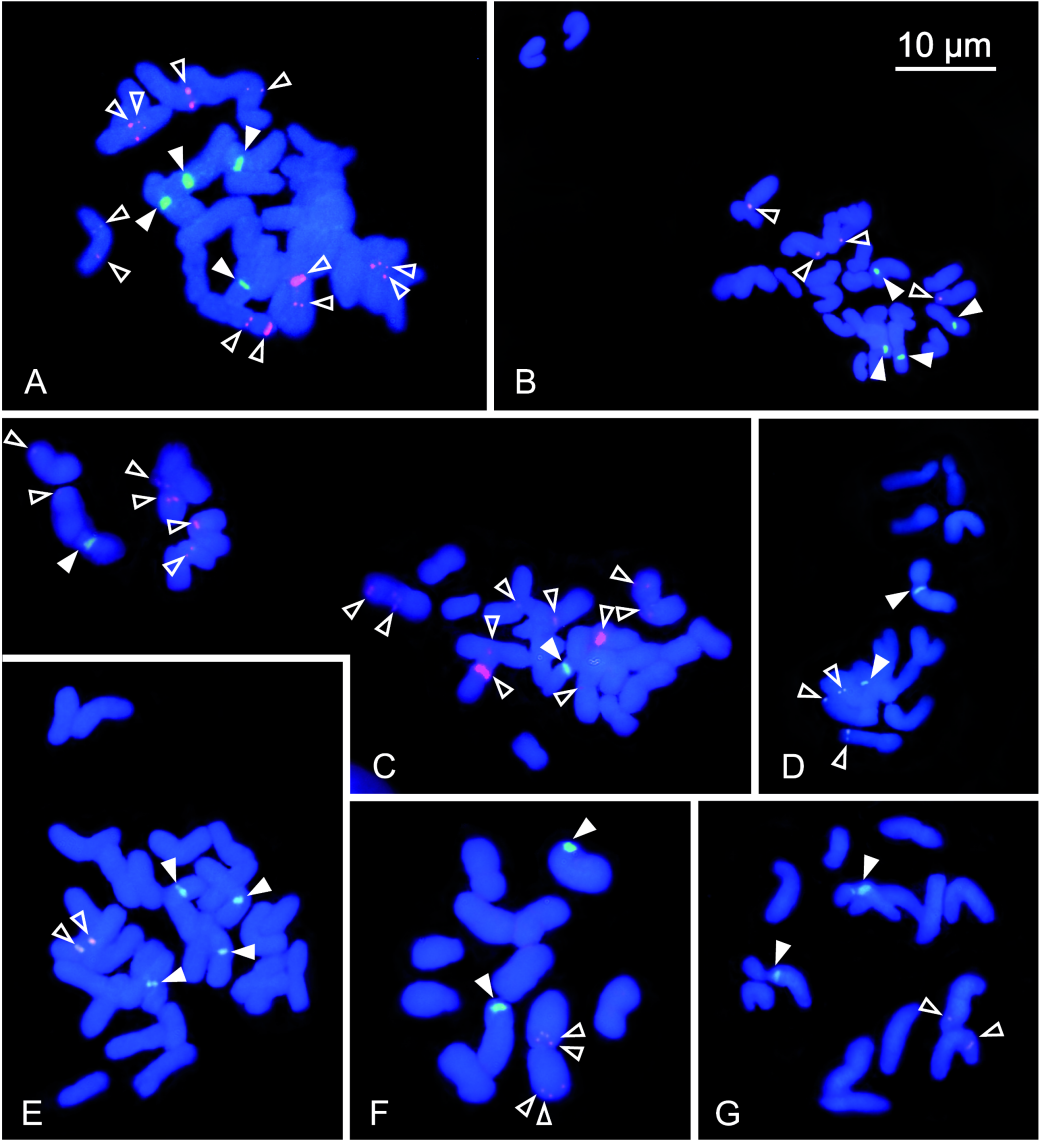
Chromosomes of *Phalaris aquatica* (A), *P*. *caroliniana* (B), *P*. *minor* (C), *P*. *coerulescens* (D), *P*. *arundinacea* (E), *P*. *canariensis* (F), and *P*. *paradoxa* (G) after *in situ* hybridisation with 5S rDNA (red signals, marked by open arrowheads), 45S rDNA (green signals, marked by filled arrowheads) and counterstaining with DAPI.

For *in situ* hybridization, the cover slip of the slides was removed with a razor blade after freezing the slides at -90°C, air-dried and treated with 100 μg/ml RNAse in 2× SSC at 37°C for 1 h (humid chamber), and washed twice in 2× SSC. Chromosomes were stabilized in 4% solution of freshly depolymerized paraformaldehyde in 10× PBS (pH 7.5) at room temperature for 10 min, washed in 2× SSC, dehydrated in an ethanol series (70, 95, 100%; 5 min each), and air-dried. The hybridization mixture consisted of 2.5 ng/μl labelled probe DNA, 10% 20× SSC, 50% deionized formamide, 20% dextran sulphate (50% w/v), 1% sodium dodecyl sulphate (SDS, 10% w/v), 2.5% blocking DNA (400 μg/ml) and H_2_O. 20 μl of the probe mixture were loaded onto each slide, and covered with a cover slip. Probe DNA and DNA of the chromosomes was denatured for 2 min at 80°C on a hotplate and hybridization was carried out overnight at 37°C in a moist chamber. After hybridization, the cover slips were loosened and the slides were immersed for 20 min in 2× SSC at 55°C, then rapidly dehydrated in an ethanol series (70, 95, 100%; 2 min each) at room temperature, and air-dried. Unhybridized DNA was made visible with the non-specific fluorochrome 4,6-diamidino-2-phenylindole (DAPI; 1.5 μg/ml) dissolved in fluorescence antifade solution (Vectashield, Vector Laboratories). Slides were covered with a cover slip, and screened with a Zeiss-Axioskop 2 epifluorescence microscope equipped with filter block 02 for detection of DAPI, 09 for fluorescein and 15 for tetramethylrhodamine fluorescence. Images were captured separately using computer-assisted cooled CCD camera (Zeiss Axiocam HRC) and subsequent overlaid employing Zeiss Axiovision software (shown in Fig 1).

### Karyotyping

**Fig 2:**
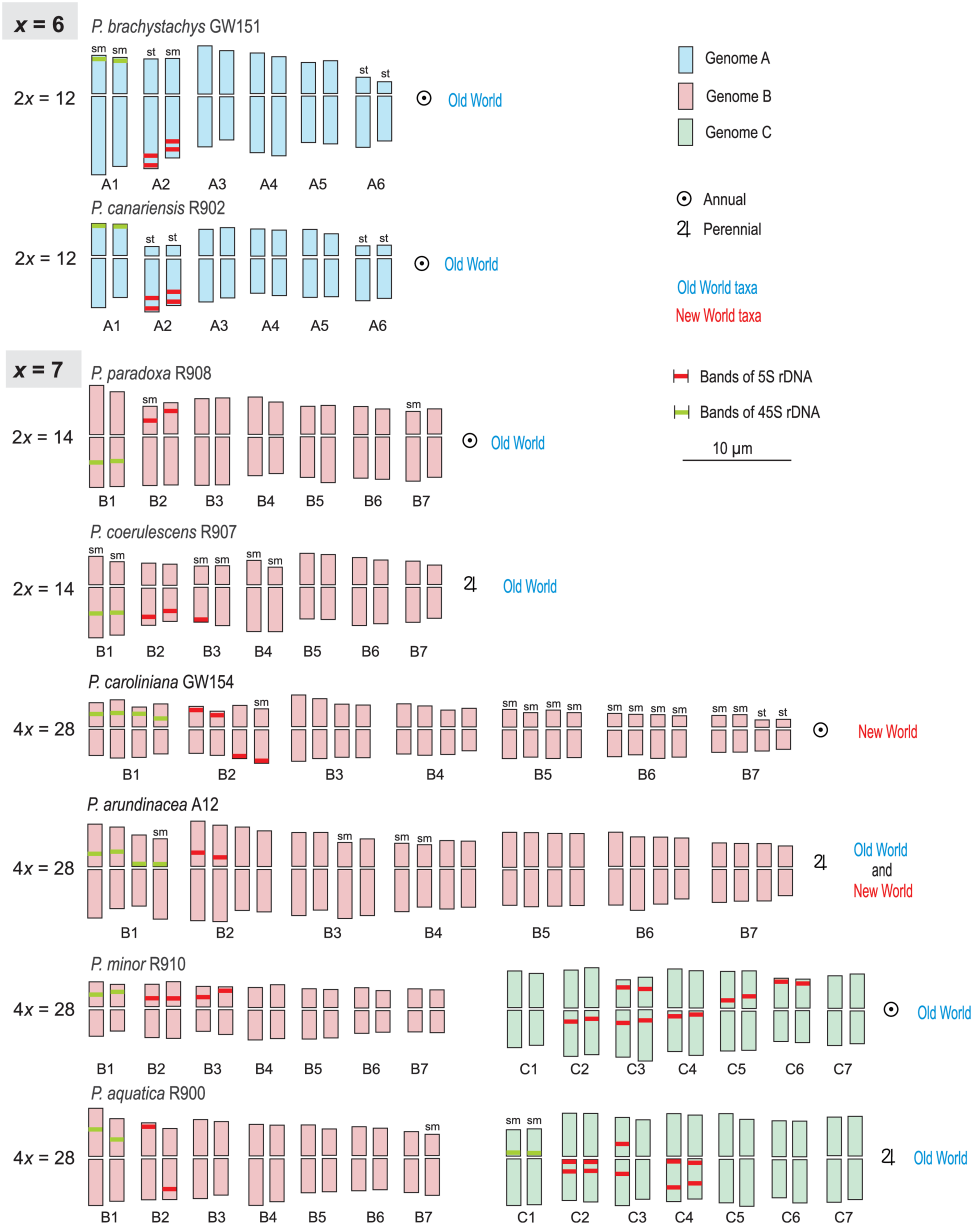
Idiograms of chromosome complements of diploid and tetraploid *Phalaris* species. Chromosome pairs are arranged into groups of presumable homologues or homoeologues according to their 45S (green bands) and 5S rDNA (red bands) probe signals and karyotype features (length and symmetry). Chromosomes were designated below rendering their affiliation to the genomes A (blue), B (red) or C (green) and the chromosome number (1, 2, 3, …). sm—submetacentric, st—subtelocentric, no sign—metacentric.

The number of metaphase plates used for karyotyping varied from 4 to 10. One karyotype was chosen for calculations shown in Table 1. Calculations of karyological parameter follows Paszko [29] and Peruzzi & Eroğlu [36]: TML = Total Monoploid Length of chromosome set; M_CA_ = Mean Centromeric Asymmetry [= Mean (L − S) / (L + S) × 100, L = total length of long arms, S = total length of short arms], CV_CL_ = Interchromosomal Asymmetry [= (sCL / xCL) × 100, sCL = standard deviation of chromosome length in a chromosome set, xCL = mean chromosome length]. Chromosome length of long and short arms, localization of constrictions, and localization of 5S and 45S rDNA sites were pictured using COREL DRAW X8 program to reconstruct a complete chromosome idiogram for each taxon. Chromosomes were designated by the letters A, B, C according to the respective genome variant combined with Arabic numbers ordered by the localization of 45S rDNA signals (1), 5S rDNA signals (2, 3 etc.) and in the following to their individual length. The terminology for chromosome shape according to the centromere position metacentric, submetacentric (sm) and subtelocentric (st) followed Levan et al. [37]. Idiograms are shown in Fig 2.

## Results

### Ploidy levels, chromosome numbers and karyotypes

**Fig 3:**
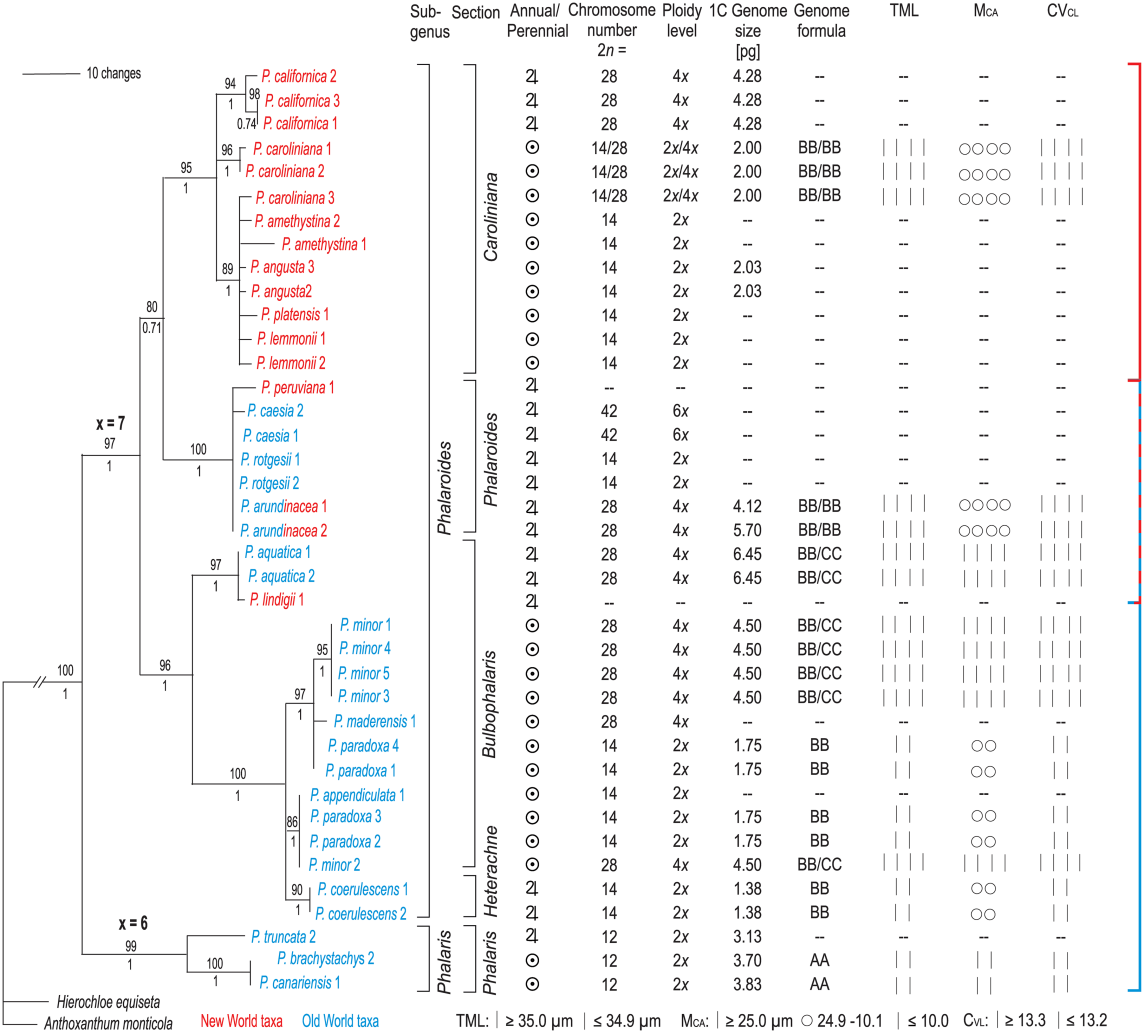
Taxonomic classification [9], life form, chromosomal properties and genome size [38] of diploid and tetraploid *Phalaris* species on a ITS phylogram based on Bayesian inference [8]. Parsimony bootstrap values and Bayesian support are noted above and below the branches.

Fig 1 shows mitotic metaphases of the taxa investigated after *in situ* hybridization with 45S and 5S rDNA and counterstaining with DAPI. The chromosome numbers observed were 2*n* = 12, 14, and 28, corresponding to basic chromosome numbers of *x* = 6 and 7 with ploidy levels of 2*x* and 4*x* (Table 1). Karyotype analyses resulted in detailed measurements and are presented in Table 1 and Fig 3. Total Monoploid Lengths (TML) varied from 28.8 μm in *P*. *caroliniana* to 50.2 μm in *P*. *brachystachys*. Interchromosomal Asymmetry (CV_CL_) was lowest in *P*. *aquatica* (CV_CL_ = 11.8) and highest in *P*. *minor* (CV_CL_ = 25.8). In the taxa with *x* = 7, Mean Centromeric Asymmetry (M_CA_) varied in taxa with x = 7 from 6,8 in *P*. *minor* to 19.3 in *P*. *coerulescens* and in the taxa with x = 6 it ranged between 25.5 in *P*. *brachystachys* and 31.4 in *P*. *canariensis*, indicating more asymmetric chromosomes in the taxa with the basic number of *x* = 6, which corresponds to their higher proportion of subtelocentric or submetacentric chromosomes (Table 1, Figs 2 and 3).

### Chromosomal distribution of 45S sites

Almost all *Phalaris* accessions had two sites of 45S rDNA per diploid chromosome set, with the exception of *P*. *minor* which had only one (chromosomes A1, B1, C1; Fig 2). Localizations of the 45S rDNA sites show some similarities between the taxa: In *P*. *canariensis* and *P*. *brachystachys* (both with x = 6) 45S rDNA signals were present in subtelomeric position in the short arms. In diploid *P*. *coerulescens* and *P*. *paradoxa* 45S bands occurred intercalary in the long arms, whereas in the tetraploid *P*. *caroliniana* and *P*. *minor* they are located intercalary in short arms. In the tetraploid *P*. *arundinacea* and *P*. *aquatica*, two of the four 45S rDNA sites are located intercalary, the other two occur proximally close to the secondary constriction, both in the short arms.

### Chromosomal distribution of 5S sites

Numbers of 5S rDNA sites varied considerably from one to eight per diploid chromosome complement. The 5S rDNA sites were situated in the chromosomes of *Phalaris* species either subtelomeric, intercalary or centromeric. *Phalaris canariensis* and *P*. *brachystachys* (both with x = 6) show common patterns in having consistently two sites of 5S rDNA distally to the secondary constriction in the long arm of subtelocentric, or submetacentric non- satellite chromosomes (chromosomes A2; Fig 2). The highest number of 5S rDNA sites was found in tetraploid *P*. *minor* with 16 sites distributed across 14 chromosomes (B2, B3, C2-C6; Fig 2), followed by *P*. *aquatica* with 12 sites within seven chromosomes (B2, C2-C4; Fig 2). They were localized mainly intercalary or near the centromere. Only one pair of *P*. *minor* (C6) and one chromosome of *P*. *aquatica* (B2) had subtelomeric 5S rDNA bands. The lowest number of 5S rDNA sites was found in tetraploid *P*. *arundinacea* with only two sites per tetraploid chromosome complement, localised intercalary in B2. Two sites per diploid chromosome set showed in diploid *P*. *paradoxa* and tetraploid *P*. *caroliniana*. They were located in *P*. *paradoxa* intercalary in the short arms of chromosomes B2, in *P*. *caroliniana* more subtelomeric either in the short arms or the long arms (B2). *Phalaris coerulescens* had three sites localised intercalary (B2) or subtelomeric (one of chromosomes B3) in the long arms.

## Discussion

We uncover the following karyotypic evolutionary trends: (1) the chromosomal mechanisms of basic number reduction from *x* = 7 to *x* = 6 within the early diverging subgenus *Phalaris* and their outcomes for karyotype, genome size, morphological traits and habit form, (2) the role of chromosomal rearrangements, contribution of three different genomes and nature of polyploidy to the evolution of the genus, and (3) the significance of chromosomal reshuffling for species diversification, reproductive isolation, speciation, and geographic distribution.

### Chromosomal mechanism of basic number reduction from *x* = 7 to *x* = 6 and its impact on karyotype

*Phalaris* comprises species with two different basic chromosome numbers of *x* = 6 and *x* = 7. Based on systematic treatments of morphological and molecular phylogenetic traits as well as on the plausibility that the most common basic number in the Aveneae-Poeae is *x* = 7, it was postulated that *x* = 7 is the ancestral basic number, from which *x* = 6 could have been derived early in their evolution [8, 19, 39].

Increase and decrease of basic chromosome number played an important role in the evolution of grasses in general [40, 41, 42, 43, 44]. Alterations in basic chromosome number may originate via modifications in mitosis, but most often result from irregularities in meiotic cell division, such as asynapsis, non-homologous recombination, loss of recombination or mis-segregation [45, 46, 47]. As consequences of these mechanisms, aneuploid or dysploid chromosome complements can originate. Aneuploidy as originally defined by Täckholm [48] refers to the gain or loss of whole chromosomes or parts of chromosomes. Hence, aneuploidy often entails basic number change, deviations in DNA content and the gain or loss of genetic information [3]. Dysploidy (called pseudoaneuploidy), on the other hand, alters the chromosome number via chromosome rearrangements such as reciprocal translocations, inversions, chromosome breakages and fusions, but does not coercively result in changes in DNA content and the gain or loss of genetic information [4, 42, 49, 50]. Therefore, dysploid chromosome rearrangements are often tolerated, while monosomic and nullisomic aneuploidy tend to be lethal in non-polyploid lineages [4].

Somatic aneuploidy is rarely detected in established plant species [4] and none of the whole genome sequencing projects from extant monocots, or some eudicots, show the removal of a whole chromosome [42, 51, 52]. Dysploid alteration was described to be the dominant mechanism of basic chromosome number reduction in grasses [42]. Reciprocal translocation was recognized as a mechanism for dysploid alteration of chromosome numbers, with two chromosomes exchanging chromosomal regions simultaneously accompanied either by ‘end-to-end fusion’ or ‘nested fusion’ of the breakpoints [50, 53]. In the first case, a chromosome undergoes symmetric reciprocal translocation with another chromosome with breakpoints close to the centromere, and close to one arm end in the other chromosome. In the latter case, also described as ‘insertional dysploidy’ [42], a whole chromosome is inserted in the centromeric region of another chromosome in a single translocation event, followed by the inactivation of one of the centromeres [42, 54].

**Fig 4:**
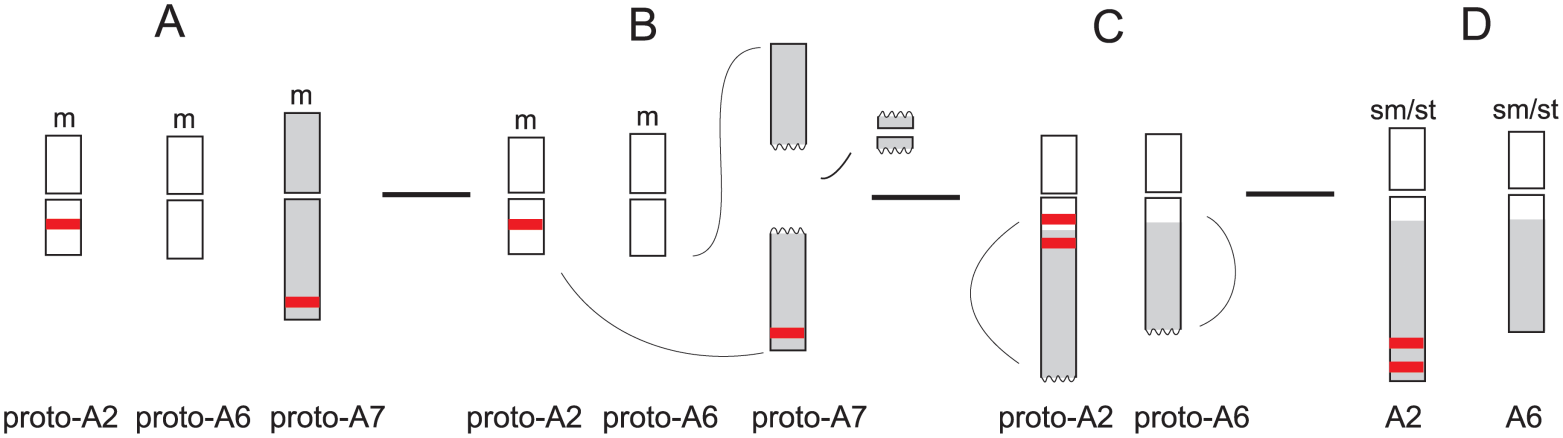
Possible scenario of reductional dysploidy in the genus *Phalaris*. A: Chromosome prototypes (proto) of a fictive ancestral *x* = 7 genome A karyotype numbered according to the ideograms of *P*. *brachystachys* and *P*. *canariensis* in Fig 2; B: Pericentromeric break in proto-A7, end-to-end fusion with proto-A2 and proto-A6 and loss of centromere; C: Paracentric inversion of fused arms; D: Reductional dysploidy to an extant *x* = 6 karyotype with strong asymmetric chromosomes. m—metacentric, sm/st—submetacentric/subtelocentric.

In our study we have scrutinized which mechanism was the cause of the decrease of the basic number in *P*. *brachystachys* and *P*. *canariensis* (2*n* = 12). Both karyotypes appear highly similar with respect to the occurrence of four subtelocentric/submetacentric chromosomes (A2/A6) and two chromosomes with two 5S rDNA sites each distal in their long arms (A2; Fig 2). These conspicuous pattern, which are not found in the *x* = 7 taxa of *Phalaris*, provide insight into a possible descending dysploidy scenario of that early diverging lineage. The results let assume, that dysploid variation was primarily caused by reciprocal translocations between an ancestral metacentric chromosome with a proximal 5S rDNA site (proto-A7 chromosome) and two further ancestral chromosomes (proto-A2 and proto-A6), one of them with 5S rDNA (proto-A2) of an ancestral chromosome complement with 2*n* = 14 (Fig 4). We propose that in the course of these process two parts of the 5S rDNA bearing chromosome (the proto-A7 chromosome) arose by a breakage in the centromeric/paracentromeric region. Subsequent loss of the centromere and symmetric reciprocal translocation of the two parts by their telomeres into breaks of telomeric regions in two other metacentric chromosomes (the ancestral proto-A2 and proto-A6) resulted in interchromosomal telomere-telomere fusion (‘end-to-end fusion’ [50]). This was followed by pericentric inversions of the translocated chromosome segments or entire arms, leading to the *x* = 6 karyotypes with two submetacentric/subtelocentric chromosomes A2 and A6, of which A2 received the 5S rDNA. The original centromere-telomere polarity of the chromosome arms is maintained in the new chromosomes.

Various trends of increasing and decreasing karyotype symmetry were described depending on the systematic group concerned [55]. In this investigation the change from symmetrical karyotypes, which are frequently considered as ‘ancient’ in many angiosperms [26], to strongly asymmetrical karyotypes is shown by *x* = 6 *P*. *brachystachys* and *P*. *canariensis* (chromosomes A2 and A6; Figs 2 and 4; see high M_CA_ values in Table 1 and Fig 3). However, the floret structures of both species *P*. *brachystachys* and *P*. *canariensis* of our study are considered as ancestral in the genus *Phalaris* [8], what means that a morphologically ancestral character state is coupled with a ‘derived’ chromosomal structure of reduced basic chromosome number and increased asymmetry. The described mechanism of reciprocal translocations leading to rather asymmetrical karyotypes in species with a derived chromosome number is not common. There have been reports of plant genera in which a lower and derived basic chromosome number is linked with more symmetrical karyotypes, e.g. in *Turnera*, which is interpreted as ‘secondary tendency’ in chromosome evolution [56].

### Genome size and the putative correlations with morphological traits and life form

In contrary to findings that genome sizes within some taxonomic groups stay within a relatively narrow range (e.g. in Brassicaceae [53]), it has been found that they could vary considerably and change rapidly in other families (e.g. in Caricaceae, Turneraceae, Poaceae, Arecaceae; [56, 57, 58, 59, 60]. Moreover, species with the lowest chromosome number, based on descending dysploidy, could have genomes that are twice as large as those of their closest relatives [57]. C-value analyses in *Phalaris* [38] show that the *x* = 6 species have a significantly higher genome size than other diploids in the *x* = 7 group (see Fig 3). Since there is no indication of polyploidy, this could indicate that the *x* = 6 taxa experienced accumulation of repetitive elements early at their split from *x* = 7 taxa. An enhanced genome size likely is due to enormous bursts in transposon amplification through the loss of repression and elimination of transposable elements [57, 61, 62]. Since transposable elements are preferentially localized near or within rDNA clusters [63, 64, 65], the translocation of chromosome segments with 5S rDNA loci could could be related to the transposon amplification in the *x* = 6 taxa. On the other hand, lower C-values in the *x* = 7 taxa could also indicate a loss of parts of the genome in the *x* = 7 taxa after the *x* = 6 taxa split.

According to Bennett [66], plants with large genomes exhibit larger morphological traits. This has been confirmed in several groups of related species [67]. Although the correlation between genome size and phenotypic dimension is often reduced when using higher phenotypic scales [68], this hypothesis is supported by the species of *Phalaris*. Within the genus, a trend in sterile lemma reduction was suggested [8], with members of the early diverging *x* = 6 lineage displaying relatively large and lanceolate sterile lemmas, followed by gradual reduction in size, culminating in almost obsolete sterile lemmas in one of the terminal *x* = 7 clades. This morphological pattern is strongly correlated with reduction in genome size.

Cytological character states appear to diversify in association with life form [40]. Decreased chromosome numbers, for example, are frequently found in short-lived annuals that grow in ephemeral habitats in semi-desert regions. Watanabe et al. [69] suggested that the change in habit from perennial to annual is correlated with dysploid reduction in chromosome number, increases in mean chromosome length and karyotypic asymmetry. According to these authors, reductions in chromosome number resulting in fewer haploid chromosomes that favour the shortening mitotic cell cycle conducive to more rapid development under the time-limited environment. Within the whole genus *Phalaris*, this correlation between genome size and life form is not obvious. The genome sizes of the annuals *P*. *brachystachys* and *P*. *canariensis*, (1C value 3.70 and 3.83 pg) are comparable to those of their closest relative in subgenus *Phalaris*, perennial *P*. *truncata* (3.13 pg). Similarly, within the *x* = 7 group (subgenus *Phalaroides*), the closely related perennial *P*. *coerulescens* (1.38 pg) and annual *P*. *paradoxa* (1.75 pg) from the *Heterachne*/*Bulbophalaris* clade have comparable genome sizes. Furthermore this pattern is also found in the *Phalaroides*/*Caroliniana* clade, in which annuals and perennials have widely corresponding genome sizes of 2–3 pg.

### Chromosome evolution inferred from chromosomal rearrangements, different genomes and polyploidy types

Chromosome rearrangements such as whole-arm reciprocal translocation, pericentric and paracentric inversion and duplication or deletion of chromosome fragments do not coercively result in changes in chromosome number in *Phalaris* and hence dysploidy, but can be useful for karyotype modeling in the genus. Thus, it seems likely that the distinction between taxonomic groups of the genus is caused by variations of repetitive DNA, changing position of telomeric, subtelomeric, intercalary or near the centromere between the long arms and the short arms in the *Phalaris* sections, which result in different inter- and/or intrachromosomal asymmetry of the chromosome sets (Table 1, Figs 2 and 3). Although it is frequently supposed that 5S rDNA sites are less variable in number and position than 45S rDNA sites [70], in this genus the 5S rDNA sites are highly variable in number and position, whereas the number of one 45S rDNA site per monoploid chromosome set keeps comparatively stable. The exception is the tetraploid *P*. *minor*, in which a loss of 45S rDNA sites is likely (Table 1, Fig 2). Additionally many investigations of plants have revealed that in spite of the wide dispersion capacity of ribosomal DNA, the number of rDNA sites tends to be restricted to two and four per diploid karyotype [34, 70]. We have found, by contrast, a conspicuous increase of the 5S rDNA sites in tetraploid species *P*. *aquatica* and *P*. *minor* of section *Bulbophalaris* (Table 1). We hypothesize that in both tetraploids this is due to the occurrence of a genome (genome C), which is not present in other species, along with the more widespread genome B.

**Fig 5:**
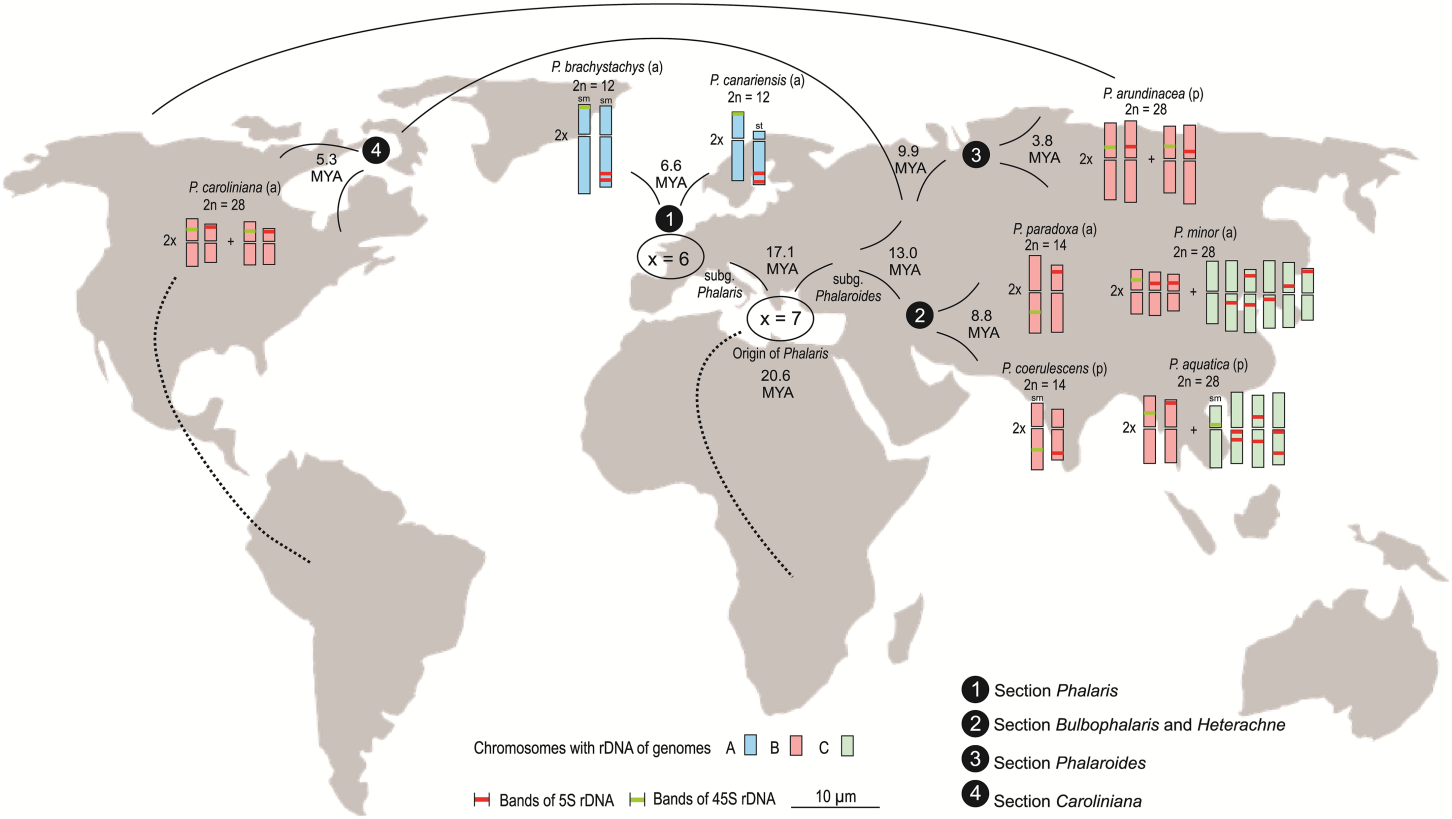
Geographical distribution of different genomes A, B, and C in eight species of *Phalaris* and possible expansions routes and time of diversification within the genus according to Voshell & Hilu [10].

Altogether, within the genus *Phalaris*, karyotypes of three genomes can be reconstructed (Figs 2 and 3): genome A with *x* = 6, occurring in Mediterranean *P*. *brachystachys* and *P*. *canariensis*; genome B with *x* = 7, occurring in all investigated taxa of subgenus *Phalaroides* (cosmopolitan); and genome C with *x* = 7, only found in tetraploids *P*. *aquatica* and *P*. *minor* (Mediterranean and Middle East). Genome A with the dysploid chromosome number shows a strongly asymmetrical karyotype due to the occurrence of four submetacentric/subtelocentric chromosomes. This genome displays one pair of short arm telomeric 45S rDNA sites and one pair of long arm subtelomeric 5S rDNA double band sites. Genome B is characterized by a more symmetrical karyotype, one pair of intercalary 45S rDNA sites and one pair of (sub-)telomeric 5S rDNA sites. Genome C karyotype has one pair of 45S rDNA and the conspicuous pattern of 5–6 pairs of 5S rDNA sites (Figs 2 and 5).

Polyploidy is one of the main processes in genome evolution in *Phalaris* [8]. Our results suggest autopolyploidy for tetraploid *P*. *caroliniana* and *P*. *arundinacea*, containing fourfold genome B and allopolyploidy (amphidiploidy) for tetraploid *P*. *aquatica* and *P*. *minor* with genomes B, and C. Polyploidy, especially if coupled with hybridization (allopolyploidy), has long been recognized as a major driver of genetic diversity. Polyploids frequently establish novel traits that are not present in their diploid progenitors, contributing to plant diversification and speciation [6, 71, 72, 73, 74, 75, 76, 77, 78, 79, 80].

Chromosomal data (Fig 2) along with previously published gene phylogenies and the interpretation of morphological traits [8] suggest that a B genome karyotype such as that found in extant *P*. *coerulescens* may be comparatively ancient and comes close to the putatively ancestral *x* = 7 *Phalaris* karyotype (Fig 4). It was the hypothetical starting point for chromosome rearrangements that led to the other karyotypes and for polyploidization (Figs 2, 4 and 5). The described fission/fusion events probably occurred in the common *x* = 6 ancestor with genome A of *P*. *brachystachys*, *P*. *canariensis* and the perennial *P*. *truncata* which was not studied here. On the other hand, the karyotype of annual *P*. *paradoxa* (genome B) was derived from the putatively ancestral *x* = 7 karyotype by loss of 5S rDNA repeats of B3 and pericentric inversion of 5S rDNA bearing chromosomes B2 (Fig 2). Tetraploid perennial *P*. *arundinacea* originated by autopolyploidy. The variation in 5S and 45S rDNA positions observed in the karyotype of *P*. *arundinacea* is likely due to a pericentric inversion in B1, B2 and the loss of one pair of 5S rDNA site in B2. Such a tetraploid karyotype is found also in annual *P*. *caroliniana* which is characterized by stronger asymmetrical chromosomes. Allopolyploids *P*. *aquatica* (perennial) and *P*. *minor* (annual) may have originated by hybridisation of diploid genome B and C taxa and subsequent polyploidization and selective suppression of 45S rDNA loci inherited from one of the progenitors in *P*. *minor* (nucleolar dominance [81]). Extant diploid genome C taxa are not yet known but judging from the tree topology in Fig 4 *P*. *lindigii* or other species of section *Bulbophalaris* would be potential candidates.

### Chromosomal reshuffling and its role for species diversification, reproductive isolation, speciation and geographic distribution

Chromosomal reshuffling and ploidy change (polyploidy or dysploidy) affect directly chromosome structure which contributes to diversification, reproductive isolation and speciation [4]. For the role of chromosome rearrangements in such speciation events, two different models have been suggested, the ‘hybrid sterility’ and ‘suppressed recombination’ model [82]. In the ‘hybrid sterility’ model, individuals which are heterozygous for the respective chromosome rearrangements, show diminished fertility which entails reproductive isolation of sympatric populations [82, 83]. In the ‘suppressed recombination’ model, genetic differentiation of overlapping populations is based on the suppression of recombination over inversion regions, which possibly will prefer accumulation of locally-adapted alleles within this region [83, 84]. Homoploid hybridization of two closely related species distinguished by distinct karyotypes may also contribute to genetic isolation of the hybrids from their parent species if backcrosses are sterile [5].

Our results might indicate a correlation between dysploid change, change of ploidy level, occurrence of different genomes and chromosomal rearrangement of ribosomal DNA sites and the change in floret structure, habit and geographical/ ecological distribution of the taxa.

Thus, a common ancestor of the genus, which originated approximately 20.6 million years ago [10], had genome B and a *x* = 7 karyotype with the preferential localisation of 5S and 45S rDNA repeats as independent sites (Figs 2 and 5). The Mediterranean basin was the most plausible centre of origin highlighted by molecular phylogenetic studies of the genus [10].

The phylogeny indicates that dysploidy change from *x* = 7 to *x* = 6 occurred in an early diverging lineage between ~17.1 and ~13.0 million years ago ([8, 10], Figs 2 and 5). The resulting *x* = 6 cytotypes (genome A) displays a lower diversification with only three extant species, *P*. *brachystachys*, *P*. *canariensis* and *P*. *truncata* (subgenus *Phalaris*). The distribution is confined to the Mediterranean and much narrower than that of the *x* = 7 cytotypes. Two species of the *x* = 6 group, *P*. *brachystachys* and *P*. *canariensis*, underwent an increase of genome size (Fig 3), followed by evolution of fewer yet larger caryopses [25, 27] and a change of life form from perennial to annual. These processes may have occurred from about ~6.6 million years ago and may support the hypothesis that dysploidy is not disadvantageous in terms of generating long-term persisting lineages [85]. However, the comparatively low number of only three dysploid species in *Phalaris* and the lower degree of geographical expansion is consistent with the hypothesis that fusion and fission events are neutral with respect to long-term diversification processes. They evoke neither substantial increase nor decrease of speciation or extinction processes, most likely because the inferred dysploid transitions typically do not necessarily entail changes in DNA content but only genome structural rearrangements [3].

In contrast, the phylogenetic sister subgenus *Phalaroides* with *x* = 7 cytotypes underwent higher species diversification (17 species) within about 13.0 million years. The development of smaller, lighter and hairy caryopses within the subgenus showed significant correlation with wider geographical distribution [8, 86, 87]. Mechanisms like auto- and allopolyploid formation, the participation of two different genomes B and C and the reshuffling of 5S and 45S rDNA sites were considered as important chromosomal rearrangements during species diversification of subgenus *Phalaroides*.

Cytogenetic data may imply that the Mediterranean perennial *P*. *coerulescens* of subgenus *Heterachne* has an ancestral form of the B genome, from which that of annual *P*. *paradoxa* of subgenus *Bulbophalaris* evolved by chromosome rearrangements. The participation of two different genomes B and C seems to be the main force of species formation within the Mediterranean tetraploid perennial *P*. *aquatica* and the Mediterranean to Middle East distributed annual *P*. *minor* of section *Bulbophalaris*. Despite some doubt about the diploid ancestor of the C genome it appears that allopolyploid formation happened about 8.8 million years ago, just after separation of *P*. *coerulescens* and *P*. *paradoxa*.

Evidence from our cytogenetics grouped the cosmopolitan and invasive perennial *P*. *arundinacea* and the North American annual *P*. *caroliniana* together, due to a common ancestor with genome B (autopolyploidy), which split about 9.9 million years ago into Old and New World species. Species with smaller genome size have been reported as more invasive [88] and growing in more extreme environments [67]. Initially, a diploid ancestor with genome B like *P*. *arundinacea* might have migrated over the Bering land bridge to western North America, where a secondary centre of diversification of primarily and presently mostly diploid, but secondarily autopolyploid *P*. *caroliniana* emerged about 5.3 million years ago. From there, speciation and geographical radiation appear to have occurred throughout the rest of North America. Subsequently, a diploid genome B ancestor emerged and diversified in the Old World about 3.8 million years ago and autopolyploidy of a genome B progenitor may have resulted in a *P*. *arundinacea* karyotype. The presence of non-invasive, ‘native’ tetraploid *P*. *arundinacea* in north-western North America [89] and *P*. *caroliniana* from North America which were found as tetraploid in this study suggests subsequent migration events of modern tetraploid individuals from the Old World during the Pleistocene via the Bering land bridge [8].
