# Correction: Karyotype evolution in *Phalaris* (Poaceae): The role of reductional dysploidy, polyploidy and chromosome alteration in a wide-spread and diverse genus

**DOI:** 10.1371/journal.pone.0195889

**Published:** 2018-04-12

**Authors:** Grit Winterfeld, Hannes Becher, Stephanie Voshell, Khidir Hilu, Martin Röser

There are errors in Fig 3, Fig 4 and Fig 5. The authors have provided corrected versions here.

**Fig 3:**
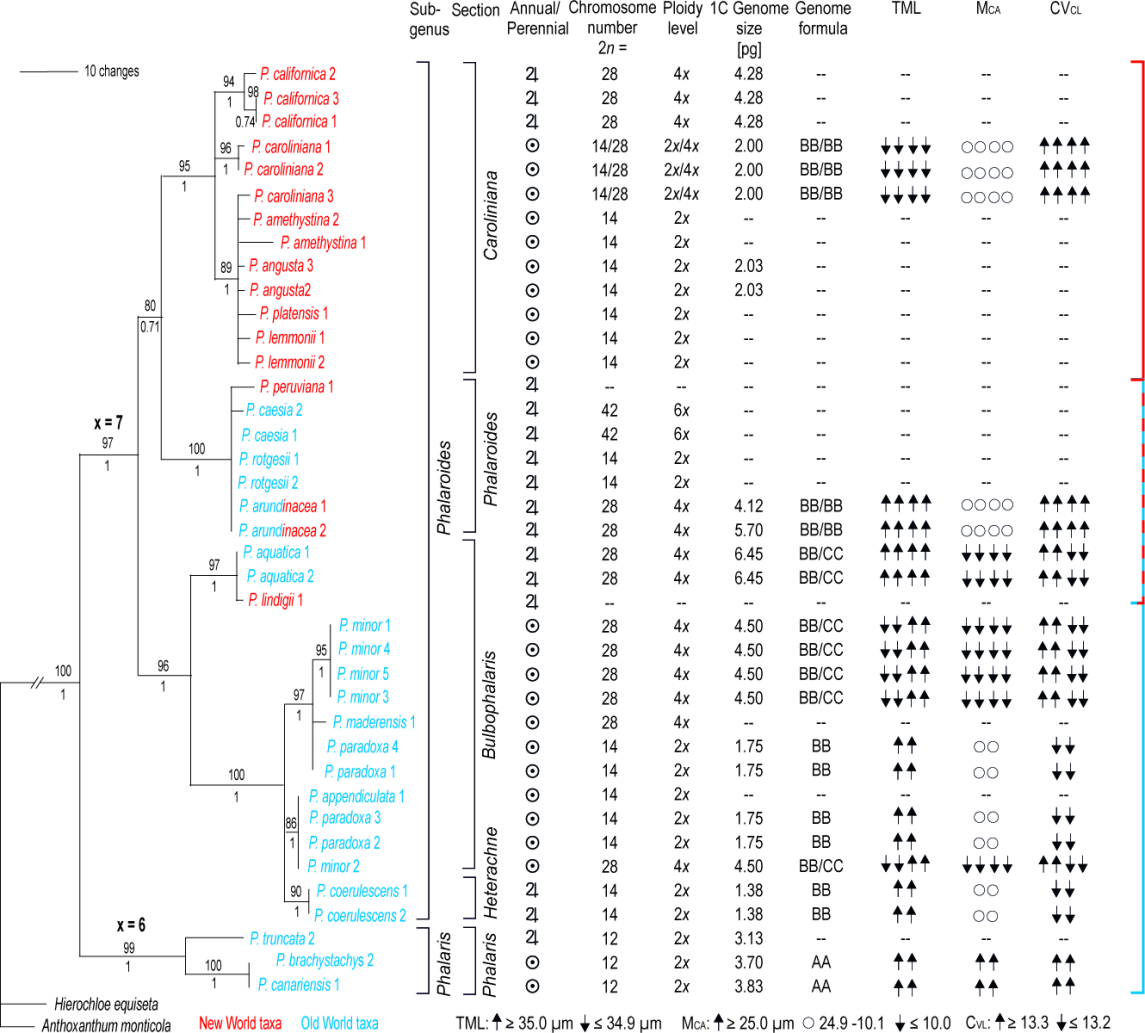
Taxonomic classification [9], life form, chromosomal properties and genome size [38] of diploid and tetraploid *Phalaris* species on a ITS phylogram based on Bayesian inference [8]. Parsimony bootstrap values and Bayesian support are noted above and below the branches.

**Fig 4:**
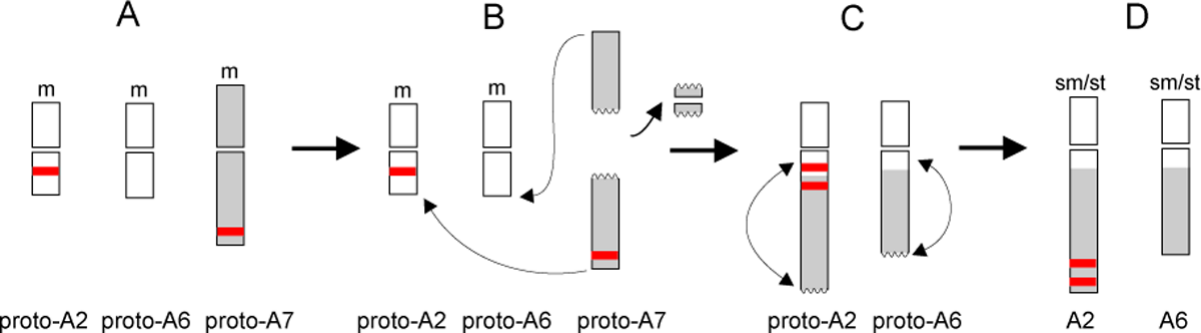
Possible scenario of reductional dysploidy in the genus *Phalaris*. A: Chromosome prototypes (proto) of a fictive ancestral *x* = 7 genome A karyotype numbered according to the ideograms of *P*. *brachystachys* and *P*. *canariensis* in Fig 2; B: Pericentromeric break in proto-A7, end-to-end fusion with proto-A2 and proto-A6 and loss of centromere; C: Paracentric inversion of fused arms; D: Reductional dysploidy to an extant *x* = 6 karyotype with strong asymmetric chromosomes. m—metacentric, sm/st—submetacentric/subtelocentric.

**Fig 6:**
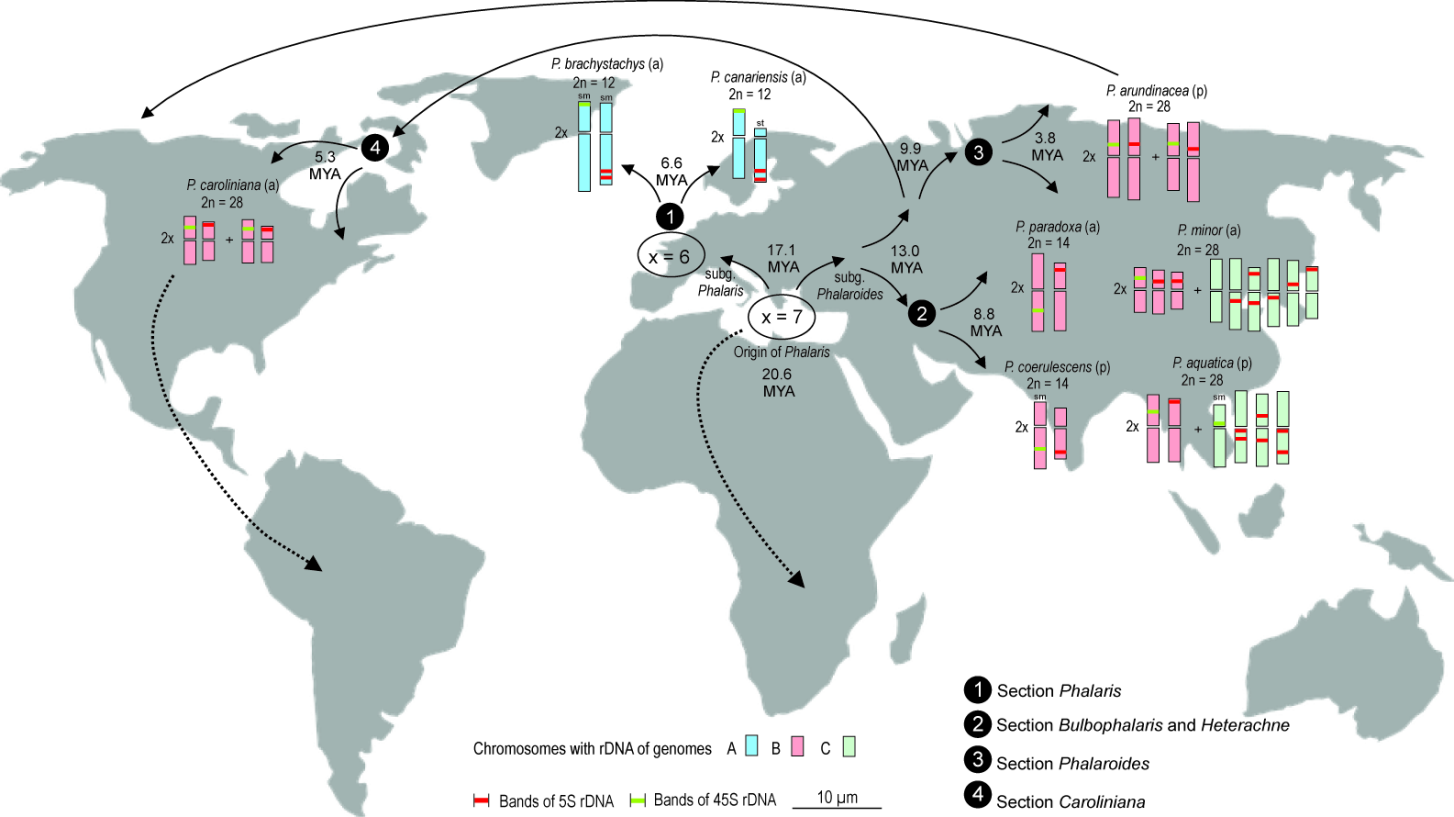
Geographical distribution of different genomes A, B, and C in eight species of *Phalaris* and possible expansions routes and time of diversification within the genus according to Voshell & Hilu [10].
